# Ogilvie’s Syndrome in a Young Female With Chronic Constipation

**DOI:** 10.7759/cureus.56557

**Published:** 2024-03-20

**Authors:** Tong Ren, Shaikh Afaq, Ali Vaziri, Olu Oyesanmi, Salman Muddassir

**Affiliations:** 1 Internal Medicine, Florida State University College of Medicine, Hospital Corporation of America (HCA) Healthcare Oak Hill Hospital, Brooksville, USA

**Keywords:** young patients, female patient, chronic constipation, acute colonic pseudo-obstruction, ogilvie’s syndrome

## Abstract

Ogilvie’s syndrome, also known as acute colonic pseudo-obstruction, is often encountered in post-surgical patients or those with serious comorbidities requiring intensive care. For this reason, it has rarely been reported in patients younger than 50 years without any predisposing risk factors. Our case report highlights a unique case of Ogilvie’s syndrome in a young female with no recent trauma or surgical history. To that extent, we discuss risk factors that predisposed her to this condition, including her history of chronic constipation. We also emphasize the need for outpatient workups for such patients to prevent the worsening of their symptoms.

## Introduction

Acute colonic pseudo-obstruction, also known as Ogilvie's syndrome, is a functional disorder characterized by acute dilatation of the colon in the absence of a mechanical obstruction. Pseudo-obstruction refers to a clinical picture mimicking mechanical bowel obstruction with signs and symptoms like abdominal distension, pain, constipation, and nausea/vomiting, but without a physical blockage [1,2]. Ogilvie's syndrome is a relatively rare event, occurring in about one out of every 1,000 inpatient admissions [3]. In over 80% of cases, Ogilvie's syndrome arises as a complication of other underlying medical conditions. A 1986 analysis of 400 cases identified the most common associated conditions: for surgical patients, trauma (11.3%) and obstetrics and gynecology (9.8%), and for medical patients, infection (10%), cardiac problems (10%), and neurological conditions (9.3%) [3]. Despite being potentially associated with severe underlying illnesses, Ogilvie's syndrome fortunately carries a low in-hospital mortality rate [4]. Risk factors include advanced age, recent surgery (abdominal, pelvic, obstetric, orthopedic spine/retroperitoneal), non-operative trauma (falls, fractures), poor functional status (Parkinson's, spinal cord injury), and a high comorbidity index [4,5]. It is most frequently diagnosed in older adults due to these factors [6]. However, Ogilvie's syndrome can also present in much younger patients with other less obvious risk factors, as highlighted in this case report [2].

The diagnosis is confirmed by a CT scan, which reveals a dilated colon with a transition zone and no obstruction. Patients are monitored closely with exams and labs to assess for complications like perforation. Initial management is conservative for mild cases, while severe cases or those failing initial treatment may require neostigmine, a reversible cholinesterase inhibitor [1]. By increasing acetylcholine levels, neostigmine can stimulate smooth muscle contraction, including in the colon [7]. This mechanism theoretically aligns with the potential benefits in cases of colonic hypomotility, as seen in Ogilvie's syndrome. In extreme cases, colonoscopic decompression is considered.

This case report describes a unique presentation of Ogilvie's syndrome in a 37-year-old Hispanic female who presented with a history of chronic constipation and severe lower abdominal pain. This case highlights the importance of considering Ogilvie's syndrome in a broader range of patients, even younger individuals without the typical risk factors.

## Case presentation

A 37-year-old Hispanic female with a past medical history of uterine leiomyoma, menorrhagia, and iron deficiency anemia presented to the emergency department with abdominal pain and constipation as her chief complaints. The patient stated that her last bowel movement was five days prior to her presentation on this visit. Per the patient, she had remained “constipated all my life” but had never undergone any medical workup. The patient was not on iron supplements and denied recent sick contact or travel. She denied any nausea or vomiting but complained of severe lower abdominal pain. The patient has no relevant personal or family medical history contributing to neuropathies and dysautonomia. Additionally, the patient has no history of chronic pain medication or narcotic use. The patient's menstrual cycle information is as follows: she was in her menstrual cycle on the day of admission, and her heavy flow consisted of three fully saturated super plus pads per day for seven days. The onset of menarche occurred at the age of 13, and the patient reports that this heavy flow is normal for her. A review of the systems was positive for heavy menstrual flow and subjective low-grade fevers. On physical examination, the patient was tachycardic at 110 beats per minute and tachypneic with a respiratory rate of 20 breaths per minute. Blood pressure and oxygen saturation remained within normal limits. The patient had hypoactive bowel sounds and was tender to light palpation throughout her abdomen. Laboratory results were significant for elevated white count and lactic acidosis. The CBC revealed microscopic anemia with a hemoglobin of 8 and a high RDW, while the CMP showed mild hypokalemia. The iron panel revealed a low iron level of less than 10. The thyroid-stimulating hormone levels were within normal ranges. Transvaginal ultrasound was significant for 5.8 cm of intramural myoma and did not appear to contribute to obstruction on imaging. CT of the abdomen showed focal wall thickening of the antrum of the stomach and proximal duodenum, mild bowel distention consistent with ileus, and liquid stool in the colon suggestive of possible gastroenteritis. The patient presented with signs of sepsis upon admission, prompting the initiation of empiric antibiotic therapy with cefepime 2g IV q12h and metronidazole 500 mg IV q8h. This choice was made due to clinical suspicion of infective colitis, which warranted broad-spectrum coverage to address potential bacterial pathogens.

**Figure 1:**
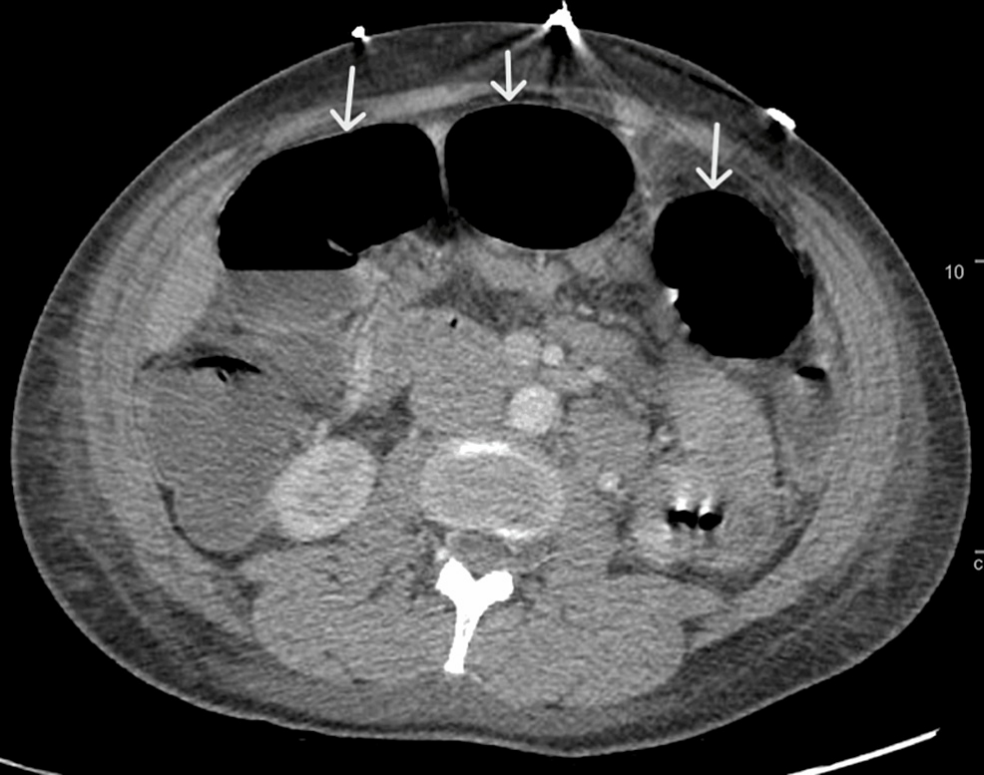
CT abdomen with contrast displayed dilatation of large bowel loops (white arrows) CT: computed tomography

During admission, the patient remained tachycardic without any resolution of her respiratory distress. Her physical examination was also significant for increasing abdominal distention, guarding, and rebound tenderness. Although the abdominal X-rays were negative for any intra or retroperitoneal free air, a repeat CT scan of the abdomen was significant for large bowel dilatation with air-fluid levels and a moderate amount of colonic fecal material (Figure 1).

By hospital day 3, the patient’s constipation had persisted despite these measures, accompanied by refractory abdominal distention. Additionally, her abdominal X-ray was significant for worsening large bowel dilatation. Due to the refractory bowel regimen and persistent large bowel dilatation in the setting of a negative infectious workup, we suspected she had developed Ogilvie’s syndrome. Given the potential role of dysautonomia in Ogilvie's syndrome, we considered the use of neostigmine. As standard clinical care, we proceeded to administer 2 mg of IV neostigmine. Prior to the administration of neostigmine in this patient, contraindications including bradyarrhythmias and bronchospasm were thoroughly assessed and ruled out. We ensured careful consideration of these contraindications and closely monitored the patient during the neostigmine infusion. Additionally, atropine was readily available at the bedside, and the patient was transferred to the intensive care unit for continuous monitoring by experienced personnel. Subsequently, the patient had two large bowel movements with a significant improvement in her abdominal distention and respiratory effort. Repeat abdominal X-rays revealed a decrease in gaseous distention of the large bowel compared to X-rays. She continued to have bowel movements daily and was able to progress to a regular diet.

As the patient's clinical condition improved and sepsis resolved, a decision was made to discontinue cefepime. This step aligns with antibiotic stewardship principles by de-escalating therapy once the need for broad-spectrum coverage diminishes. Subsequently, the patient's antibiotic regimen was transitioned to oral amoxicillin/clavulanate potassium (875 mg) twice daily for one week. It is important to note that despite comprehensive testing, including assessments for *Clostridium difficile*, *Campylobacter*, and Shiga toxin, no infectious etiology was identified. Furthermore, cultures yielded negative results. Consequently, in the absence of evidence supporting an ongoing infection, the decision was made to discontinue antibiotics altogether. Prior to discharge, she was placed on probiotics and a bowel regimen: senna glycoside 8.6 mg two tablets daily, docusate sodium 100 mg BID, and polyethylene glycol 3350 17g BID as needed for constipation. While the patient wasn't able to return for a planned follow-up due to a lack of insurance and limited resources, we did reach out to her by phone. She reported that her symptoms have resolved and she is no longer experiencing intermittent issues.

## Discussion

Ogilvie’s syndrome is characterized by spontaneous massive dilatation of the cecum and proximal colon. It is most commonly observed in postsurgical patients who have serious underlying orthopedic or neurologic disease after trauma, medical patients on ventilator support, opioids or anti-cholinergic agents, and electrolyte imbalances that can halt the enteric nervous system [4-6]. Given the aforementioned etiology, it is not surprising that Ogilvie's syndrome is usually diagnosed in an older population [6]. While conservative treatment is successful in more than 80% of patients diagnosed with the syndrome, worsening distention or abdominal tenderness should allude the clinician to a more aggressive treatment to avoid the potential risk of abdominal perforation [8]. Our patient was treated with IV neostigmine due to her refractory abdominal distension, which had persisted for more than three days. The patient tolerated the intervention well, and there were no complications. Repeat abdominal imaging showed decreased distention of the colon followed by resolution of constipation amid a significant improvement on abdominal examination.

One possible explanation for our patient could be her history of chronic constipation, which may have disrupted her enteric nervous system. This history of chronic constipation may be primarily because of colonic inertia (for which we recommended that the patient undergo further outpatient workup) [9]. It is important to note that the patient had not been on iron supplements, eliminating the possibility of adverse effects such as constipation commonly associated with their use. This absence of iron supplementation contributes to the understanding of the patient's medical history in relation to Ogilvie's syndrome. While irritable bowel syndrome could not be excluded as a cause of chronic constipation, she denied any pain, bloating, or intestinal cramping between bowel movements and denied any diarrhea. Additionally, while an outpatient workup for colonic inertia is required, obstruction of the bowel from uterine leiomyoma may also result in chronic constipation in this setting. We did not identify any studies in the peer-reviewed literature that reported Ogilvie’s syndrome in young adults with the above-mentioned past medical history. Had our patient been evaluated for the etiology of her chronic constipation earlier, this hospitalization could have easily been prevented. To that extent, we recommend clinicians recognize that chronic constipation without proper evaluation and treatment could result in Ogilvie’s syndrome, even in a much younger patient.

Differential diagnosis

Ogilvie's syndrome can be difficult to diagnose because it can mimic other conditions that cause intestinal obstruction. Table 1 compares Ogilvie's syndrome with some of the more common conditions that can cause similar symptoms.

**Table 1:** Differential diagnosis of Ogilvie's syndrome

Diagnosis	Key Clinical Features	Imaging Studies	Differentiating Points from Ogilvie's Syndrome	References
Acute Mechanical Bowel Obstruction	Abdominal pain, distention, vomiting, obstipation	Imaging reveals mechanical obstruction on plain films or CT scans	Presence of mechanical obstruction on imaging	[1,2]
Toxic Megacolon	Fever, leukocytosis, bloody diarrhea	Imaging may show signs of colitis, thumbprinting	Presence of colitis and bloody diarrhea	[3]
Chronic Constipation	Infrequent bowel movements, hard stools	Colonoscopy may reveal fecal impaction	No acute abdominal distention or vomiting	[4]
Fecal Impaction	History of constipation, abdominal pain, rectal fullness	X-ray confirms fecal impaction	History of constipation and confirmation of fecal impaction on X-ray	[4]
Colorectal Cancer	Change in bowel habits, blood in stool, weight loss, iron deficiency anemia	Colonoscopy reveals mass or stricture	Presence of mass or stricture on colonoscopy	[5]

## Conclusions

Our case underscores Ogilvie's syndrome in a young patient with chronic constipation. Despite its association with older populations, neostigmine intervention successfully resolved symptoms. The case emphasizes that proactive evaluation of chronic constipation's etiology could prevent hospitalization. Clinicians must recognize Ogilvie's possibility in young patients with chronic constipation, emphasizing early identification and management for prevention.
